# TREM2 deficiency exacerbates tau pathology through dysregulated kinase signaling in a mouse model of tauopathy

**DOI:** 10.1186/s13024-017-0216-6

**Published:** 2017-10-16

**Authors:** Shane M. Bemiller, Tyler J. McCray, Kevin Allan, Shane V. Formica, Guixiang Xu, Gina Wilson, Olga N. Kokiko-Cochran, Samuel D. Crish, Cristian A. Lasagna-Reeves, Richard M. Ransohoff, Gary E. Landreth, Bruce T. Lamb

**Affiliations:** 1 Department of Neurosciences, The Cleveland Clinic Lerner Research Institute, Cleveland, OH USA; 2 Kent State University, School of Biomedical Sciences, Kent, OH USA; 3 Indiana University School of Medicine Stark Neuroscience Research Institute, Indianapolis, IN USA; 4 Department of Neurosciences, Case Western Reserve University, Cleveland, USA; 5 Department of Neurosciences, Northeastern Ohio Medical University, Rootstown, OH USA; 6 Department of Neurosciences, The Ohio State University, Columbus, OH USA; 7 Biogen IDEC, Boston, MA USA

**Keywords:** TREM2, Tauopathy, Alzheimers disease, Inflammation, Immunity

## Abstract

**Background:**

Genetic variants of the **T**riggering **R**eceptor **E**xpressed on **M**yeloid Cells-**2** (TREM2) confer increased risk of developing late-onset Alzheimer’s Disease (LOAD) and other neurodegenerative disorders. Recent studies provided insight into the multifaceted roles of TREM2 in regulating extracellular β-amyloid (Aβ) pathology, myeloid cell accumulation, and inflammation observed in AD, yet little is known regarding the role of TREM2 in regulating intracellular microtubule associated protein tau (MAPT; tau) pathology in neurodegenerative diseases and in AD, in particular.

**Results:**

Here we report that TREM2 deficiency leads to accelerated and exacerbated hyperphosphorylation and aggregation of tau in a humanized mouse model of tauopathy. TREM2 deficiency also results, indirectly, in dramatic widespread dysregulation of neuronal stress kinase pathways.

**Conclusions:**

Our results suggest that deficiency of microglial TREM2 leads to heightened tau pathology coupled with widespread increases in activated neuronal stress kinases. These findings offer new insight into the complex, multiple roles of TREM2 in regulating Aβ and tau pathologies.

**Electronic supplementary material:**

The online version of this article (10.1186/s13024-017-0216-6) contains supplementary material, which is available to authorized users.

## Background

The role of innate immunity in the pathogenesis of neurodegenerative diseases remains uncertain despite intense, sustained investigation. Recent genetic, genomic and animal model studies have highlighted the importance of several innate immune molecules in the progression of many major neurodegenerative disorders, including Alzheimer’s Disease (AD), the most prevalent neurodegenerative disease worldwide [1–3]. Of particular note, in late 2012, two independent groups identified coding alterations in the ***T*** *riggering* ***R*** *eceptor* ***E*** *xpressed on* ***M*** *yeloid Cells-* ***2*** (*TREM2*) gene that dramatically increased the risk of developing late-onset Alzheimer’s Disease (LOAD) [4, 5].

TREM2 is a single-pass transmembrane glycoprotein that is expressed on myeloid lineage cells, and modulates innate immune function [6]. In the CNS, TREM2 mRNA is expressed by microglia, although the protein is not readily detected either by immunohistochemistry nor flow cytometry [7–12]. Recessive, loss-of-function mutations in *TREM2* are the genetic basis of Nasu-Hakola disease, characterized by abnormal bone cysts and early onset dementia [13–16]. TREM2 coding variants have also been associated with increased risk for amyotrophic lateral sclerosis (ALS) and frontotemporal-lobar dementia (FTLD) [17–19]. Taken together, these data suggest that TREM2 likely plays a critical role in regulating myeloid cell function within the brain, thereby modulating risk for neurodegenerative diseases. Despite this abundance of genetic validation, the pathways linking TREM2 to neurodegenerative disease have yet to be identified and strategies for therapeutic modulation are not evident.

Recent studies examined the role of TREM2 in regulating disease pathogenesis in mouse models that develop the extracellular deposits of β-amyloid (Aβ) characteristic of AD. Our studies revealed high expression of TREM2 in macrophages associated with Aβ deposits and that TREM2 deficiency virtually abrogated accumulation of these macrophages accompanied by reduced levels of several cytokine mRNAs [20, 21]. Furthermore, we reported that TREM2 deficiency leads to altered Aβ pathology that varies as a function of disease progression in APP-PS1 mice [22]. Moreover, the plaque associated TREM2+ cells expressed markers indicating their derivation from peripheral macrophages. The dramatic decrease of plaque-associated macrophages was reported by all groups studying amyloid pathology in TREM2-deficient mice, although there were discordant observations with regard to the origin of TREM2+ cells as well as the effects of TREM2 deficiency on Aβ deposition [23, 24] which have subsequently been resolved [22].

Here we address the effect of TREM2 deficiency on another cardinal aspect of neurodegeneration, intracellular neuronal aggregates of hyperphosphorylated microtubule associated protein tau (MAPT; tau), using the hTau mouse model of tauopathy. These studies demonstrate that TREM2 deficiency leads to exacerbated and earlier onset of tau phosphorylation and aggregation coupled with a profound dysregulation of stress related kinase pathways. These results suggest that myeloid cell TREM2 plays a protective role in regulating or suppressing neuronal tau pathologies and potentially implicates TREM2 signaling pathways within discrete myeloid cell populations in linking Aβ and tau pathologies.

## Methods

### Animal models

hTau^+/−^;mTau^−/−^ were acquired from the Jackson Laboratory which express all six isoforms of the human tau protein under the control of the endogenous human *MAPT* promoter, and backcrossed into *Trem2* ^*−/−*^ mice [20] to generate hTau^+/−^;mTau^−/−^:*Trem2* ^*−/−*^ and hTau^+/−^;mTau^−/−^ control mice. These mice were backcrossed for 4 generations, and maintained on a B6 background. All experiments were preformed and repeated using multiple cohorts of mice, and each individual assay was performed in duplicate. C57BL6/j mice (B6) obtained from the Jackson Laboratories were used side-by-side with *Trem2* ^*−/−*^ for control experiments.

Mice were housed in the Cleveland Clinic Biological Resources Unit and the Jackson Laboratory, facilities fully accredited by the Association and Accreditation of Laboratory Animal Care. All experimental procedures were approved by the Institutional Animal Care and use Committee at each respective institution.

### Western blotting

Mice were deeply anesthetized and perfused with ice-cold PBS, and their brains were removed, snap frozen, and stored at −80°C until use. Cortices and hippocampi were microdissected and homogenized in ice-cold T-per homogenization buffer with added protein phosphatase inhibitor and protease inhibitor cocktails (1:100) for 60 s. Homogenates were sonicated, centrifuged, and total protein concentrations assessed in tissue samples using the Bicinchoninic Acid (BCA) assay, following manufacturer’s instructions (Thermo Fisher, Cat# 23225). Absorbance values were read at 562 nm on a SpectraMax 340 PC plate reader (Molecular Devices, Sunnyvale, CA) using SoftMax Pro 5.2 analytical software. Proteins were denatured at 95°C for 15 min in 35% denaturing buffer containing LDS sample buffer (Life Technologies) and reducing agent (Life Technologies) 30–60 μg total protein were loaded along with 5 μg Magic Mark XP protein ladder (Life Technologies) onto Novex 4–12% Bis-Tris gels (Life Technologies), run at 160 V for 30–60 min, and transferred onto PVDF membranes (EMD Millipore) in 1XTAE buffer at 100 mA overnight at room temperature. After transfer, membranes were blocked with Odyssey Blocking Buffer in PBS (LI-COR Biosciences) for 1 h at room temperature and incubated in the appropriate primary antibodies in blocking buffer with added 0.1% Tween 20 overnight at 4 °C with shaking AT8 (1:5000; Pierce), AT180 (1:2000; Pierce), Tau5 (Thermo Scientific), PHF-1 (1:10,000; a generous gift from Dr. Peter Davies), MC1 (1:1000; a generous gift from Dr. Peter Davies), JNK (1:5000) and pJNK (Thr183/Tyr185; 1:1000) (Cell Signaling Technologies), ERK1/2 (1:5000) and pERK1/2 (Thr202/Tyr204; 1:2000) (Cell Signaling Technologies), GSK3β (1:5000) and pGSK3β (Ser9;1:2000) (Tyr216 1:2000) (Cell Signaling Technologies; ABCAM), p38 MAPK (1:2000) and pP38 MAPK (1:1000) (Cell Signaling Technologies), GAPDH (1:10,000; EMD Millipore) and β-Actin (1:10,000; Thermo Scientific). Membranes were washed with PBST (0.1% Tween 20), incubated for 1 h with the appropriate IR-conjugated secondary antibody, and visualized using an Odyssey IR scanner (LI-COR Biosciences) system. ImageStudio software (LI-COR Biosciences) was used for densitometric analysis and each experimental sample was normalized to total tau (Tau5), GAPDH or β-Actin.

### Sarkosyl extractions

Remaining detergent insoluble pellets prepared for Western Blotting were weighed and homogenized in 10 vol buffer H for 60 s followed by 15 s sonication at 20% amplitude, as performed in (Bhaskar, Korneth et al. 2010). Centrifuged sample supernatants were adjusted to 1% with 10% *v*/v sarkosyl solution with 1% β-ME. Sarkosyl solutions were incubated for 2 h at 37°C with agitation. Samples were then ultra-centrifuged at 150,000 g for 35 min. Sarkosyl soluble and insoluble fractions were isolated for analysis. Following BCA assay, 60 μg total protein from each fraction were denatured and reduced, run onto gels, transferred, and visualized according to our Western Blotting protocol. Micrographs contain 2 representative samples per genotype, per time-point.

### Immunohistochemistry

30 μm free floating tissue sections were prepared for standard immunohistochemistry or immunofluorescence. Standard antigen retrieval via boiling at 95 °C for 10 min in sodium citrate buffer (10 mM) was used before probing with the appropriate primary antibody, AT8, AT180, Iba1, NeuN, CD45, F4/80 diluted at 1:500, or pJNK, pERK, and pGSK3β diluted at 1:100. Secondary antibodies (1:500) conjugated to either biotin (immunohistochemistry; Vector Labs) or Alexa Fluor dyes (for immunofluorescence), or incubated with Avidin: Biotinylated enzyme Complex (ABC; Vector Labs) reagent for 1 h at room temperature. Signals were revealed by developing sections in 3,3′-diaminobenzidine (DAB with/without Ni enhancement; Vector Labs) for immunohistochemistry, or visualization using fluorescent microscopy following mounting with Permount (Fisher Scientific) or DAPI with Hardset (Vector Labs). Quantitation of tau positive cells was performed on a total of 2 medial tissue sections from 4 mice per genotype. Representative sections 200 μm × 2 mm from the apex of the frontal cortex to the white matter were utilized to count Individual p-Tau^+^ cells in layers II-III as well as IV-VI. % area quantifications were performed using 2mm^2^ regions of interest across lamina II-III within the medial frontal cortex. Regions were thresholded and quantified using ImageJ particle counter. Data are represented as mean +/− standard error. For microglial morphology analysis, a total of 20–30 microglia were selected randomly from frontal cortex and CA3 region of the hippocampus from each genotype. Individual cells were thresholded and analyzed for soma area and total cell area. Thresholded cells were then skeletonized and analyzed using ImageJ AnalyzeSkeleton plugin which outputs total number of branches along with branch junctions. 2-way ANOVA with Sidek multiple comparison correction was utilized to compare differences between genotypes, and among brain region.

### Quantitative RT-PCR (qRT-PCR)

Mice were perfused with PBS, and their brains were removed, snap frozen, and stored at −80 °C until use. Tissue was homogenized in 1% NP-40, 0.5% sodium deoxycholate, 0.1% SDS, and 1:100 protease inhibitor cocktail in PBS. RNA was isolated using chloroform extraction and was purified using Purelink (Life Technologies). cDNA was prepared from 1.5 μg using a QuantiTect Reverse Transcription kit (QIAGEN), and real-time PCR was performed for 40 cycles with the StepOne Plus real Time PCR system (Life Technologies). All primers and TaqMan probe were purchased from the Life Technologies database. Relative gene expression was determined using the ∆∆C_T_ method. ANOVA with Bonferroni or Sidak multiple comparison adjustment were used to compare B6 and hTau;*Trem2* ^*+/+*^ mice and 6 and 12-months.

### Cytokine multiplexing

Tissue was extracted and processed as described above for western blotting. Cytokine levels were all normalized to total protein concentration following BCA assay. Multiplex assays were conducted according to manufacturer’s instructions using reagents provided with the kit (Invitrogen Mouse 20-plex Cytokine Panel, Cat# LMC0006M). Following sample incubation, plates were washed with a mild detergent solution using a magnetic 96-well separator (Invitrogen Cat# A14179) three times (1 min per wash). After 30-min streptavidin-rpe incubation, plates were again washed three times followed by a final addition of 125 μl wash solution to all wells. Plates were read on a Luminex Magpix unit (Life Technologies) and initial analyses were performed by Xponent software and results exported into Microsoft Excel for further processing. Sample size was set to 50 μl and minimum count was set to 100 events/bead region.

### Statistical analysis

Data are presented as mean ± SEM unless otherwise noted. Comparisons between two groups were analyzed using Student’s *t* test (two-tailed; unpaired) at 95% confidence interval. Multiple group comparison, or multiple comparisons were analyzed using ANOVA or 2-way ANOVA followed by Bonferroni or Sidak post hoc test. Analysis was performed using Prism GraphPad or SPSS software. Significance was determined at *P* < 0.05 *, *P* < 0.01 **, and *P* < 0.001 ***. Each experiment was performed in duplicate, validated, and experimental mice were derived from multiple cohorts. Quantification was performed with the researcher blinded to experimental group and genotype.

## Results

### TREM2 deficiency worsens tau pathology in hTau mice

**Fig. 1:**
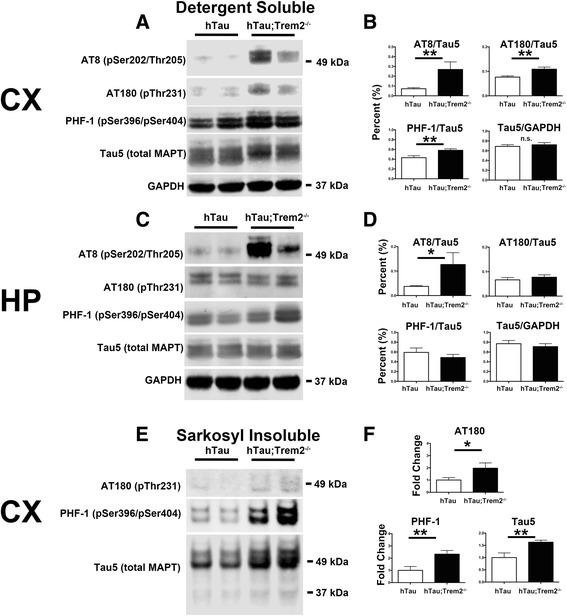
Increased soluble and insoluble tau phosphorylation in TREM2 deficient mice. **a-d** Microdissected cortices and hippocampi from hTau (*Trem2* ^*+/+*^) and hTau;*Trem2* ^*−/−*^ mice were analyzed using western blot with antibodies against AT8, AT180, PHF-1 phospho-epitopes and Tau5 (total tau). **b** Quantification of cortex western blot data revealed highly significant increases in the ratio of phosphorylated AT8, AT180, and PHF-1 to total tau but importantly, no increases were detected in total tau (Tau5). **d** Quantification of hippocampus western blot data reveals significant hyperphosphorylation at the AT8 epitope. **e** Sarkosyl extractions were performed on cortical tissue lysates from hTau (*Trem2* ^*+/+*^) and hTau;*Trem2* ^*−/−*^ mice and protein levels analyzed using western blot. **f** Quantification of western blot optical densities revealed significant increases in AT180 and highly significant increases in PHF-1 and Tau5 (total tau). All experiments used *n* = 4–6 (equal males and females) mice per group unless otherwise noted. At least two independent experiments were performed for each analysis. Error bars represent SEM. *, *P* < 0.05, **, *P* < 0.01, ***, *P* < 0.001

Humanized tau mice (hTau) lack the endogenous mouse *Mapt* gene (m*Mapt* ^*−/−*^), but instead express the full-length human *MAPT* gene driven under the endogenous human *MAPT* promoter [25, 26]. hTau mice develop age-related hyperphosphorylation, aggregation and mislocalization of tau, and exhibit modest behavioral abnormalities with age. To explore the effect of TREM2 deficiency on tau pathology and microglial mediated inflammation, hTau mice were mated to *Trem2* ^*−/−*^ mice to generate hTau;*Trem2* ^*+/+*^ and hTau;*Trem2* ^*−/−*^ mice [20, 26]. Importantly, hTau mice develop hyperphosphorylated tau around 3 months of age, which leads to the formation of insoluble tau aggregates beginning at approximately 6 months of age [25, 26]. To determine the relative levels of soluble phosphorylated tau in TREM2 deficient hTau mice at 6 months of age, detergent soluble protein extracts from microdissected cortices and hippocampi were analyzed via Western blotting. Although the level of total tau was not different between hTau/*Trem2* ^*+/+*^ and hTau;*Trem2* ^*−/−*^ mice, there were highly significant increases in tau phosphorylation at Ser202/Thr205 (AT8; *P* < 0.01), Thr231 (AT180; P < 0.01), and Ser396/Ser404 (PHF-1; P < 0.01; Fig. 1a, b) in the cortices, along with early pathogenic tau conformation specific epitope MC1 (Additional file 1). Hippocampal AT8 phosphorylation was also significantly increased in hTau;*Trem2* ^*−/−*^ mice (Fig. 1c, d), but not in other tau epitopes examined.

Next, to determine whether there was altered tau aggregation in TREM2 deficient hTau mice in addition to increases in soluble tau phosphorylation, western blots were performed on sarkosyl extracted cortical fractions [27]. We detected a significant increase in insoluble tau phosphorylated at Thr231 (AT180; *P* < 0.05) and Ser396/Ser404 (PHF-1; *P* < 0.01) as well as total sarkosyl insoluble tau (P < 0.01) in TREM2 deficient hTau mice (Fig. 1e, f). These data suggest that in addition to increases in tau phosphorylation, hTau;*Trem2* ^*−/−*^ mice exhibit enhanced tau aggregation.

**Fig. 2:**
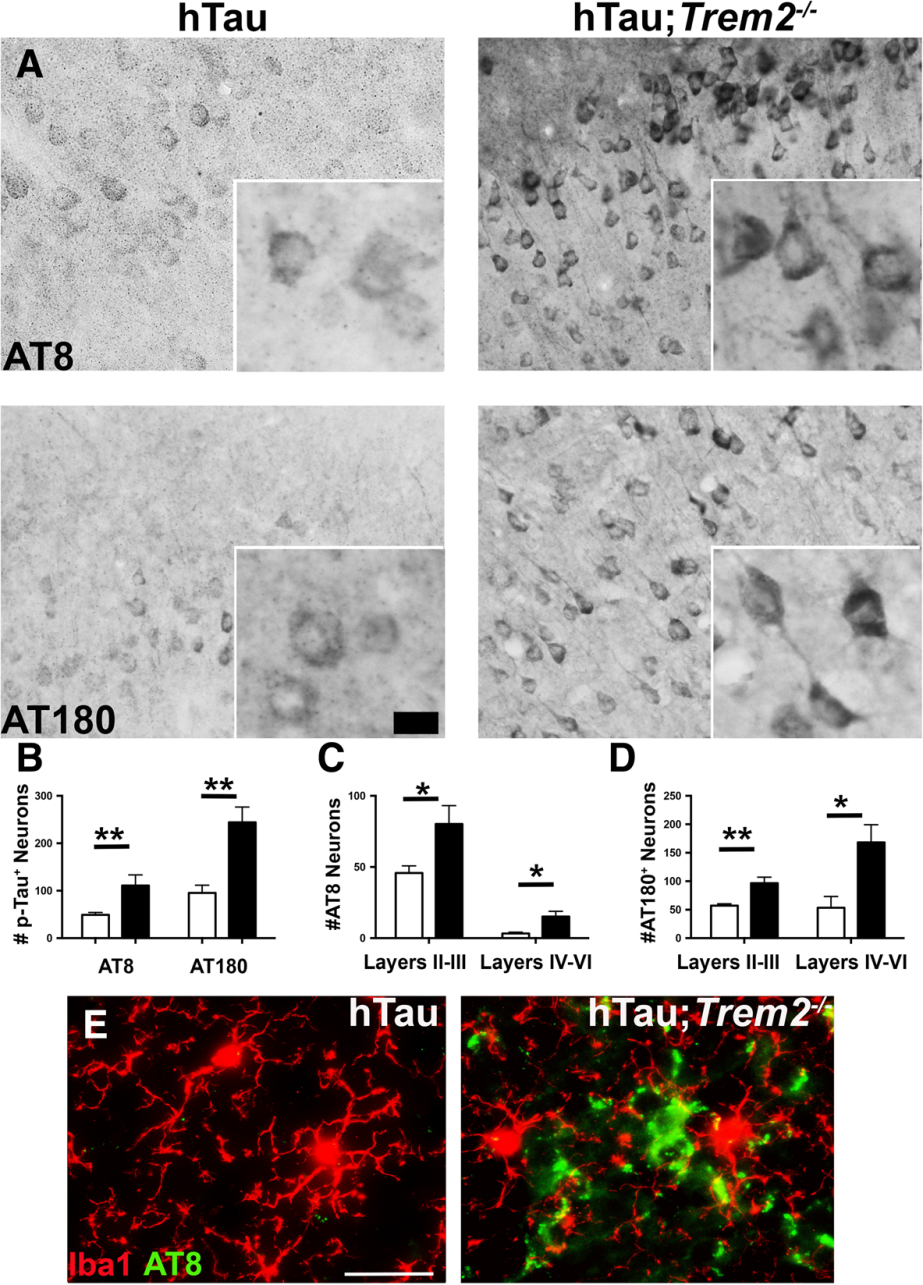
Immunohistochemistry reveals increased tau hyperphosphorylation in TREM2 deficient hTau mice. **a** Immunohistochemistry was performed on 6-month hTau (*Trem2* ^*+/+*^) and hTau;*Trem2* ^*−/−*^ mice using antibodies against AT8 and AT180 tau phospho-epitopes and revealed dramatically increased AT8 and AT180 staining in the cortex. **b** Quantitation of total p-Tau^+^ neurons revealed highly significant increases in total AT8^+^ and AT180^+^. **c, d** Laminar specific p-tau^+^ counts reveal significant increases in specific layers II-III in AT8^+^ and AT180^+^ neurons in hTau;*Trem2* ^*−/−*^ mice. Additionally, robust increases in layers IV-VI were detected in hTau;*Trem2* ^*−/−*^ mice. **e** Immunofluorescent co-labeling was performed with AT8 and macrophage/microglia specific Iba1 revealing reactive microglia in direct association with neurons heavily laden with phosphorylated tau in hTau;*Trem2* ^*−/−*^ mice compared to hTau controls. All experiments used n = 4–6 (equal males and females) mice per group unless otherwise noted. At least two independent experiments were performed for each analysis. Bars, **a**-30 μm, **e**-100 μm

To examine the cellular localization of phosphorylated tau species, immunohistochemistry with antibodies directed against the various tau phospho-epitopes was performed. In support of our biochemical analyses, significantly increased Ser202/Thr205 (AT8) immunoreactivity (IR) was observed in hTau;*Trem2* ^*−/−*^ mice when compared to hTau;*Trem2* ^*+/+*^ controls (Fig. 2a-c; Additional file 2). This increased IR was localized within neuronal soma, axons and dendrites throughout cortical layers II-III, with modest increases in layers IV-VI (Fig. 2a) in hTau;*Trem2* ^*−/−*^ mice. Furthermore, significantly increased Thr231 immunoreactivity (AT180) was also evident in layers II-III but was robustly increased within deeper cortical layers IV-VI (Fig. 2a–d). Fluorescent co-labeling with anti-AT8 and Iba1 revealed reactive microglia juxtaposed to regions laden with phosphorylated tau in hTau;*Trem2* ^*−/−*^ mice suggesting a potential role of microglia in mediating the tau phenotypes observed in hTau;*Trem2* ^*−/−*^ mice (Fig. 2e; Additional file 1). No significant differences were noted in p-tau immunoreactivity within hippocampi between hTau and hTau;*Trem2* ^*−/−*^ mice. Importantly, these increases in phosphorylation were not observed in other controls including 3 month hTau and hTau;*Trem2* ^*−/−*^ mice (Additional file 3) and age-matched non-transgenic *Trem2* ^*+/+*^ mice and *Trem2* ^*−/−*^ controls (Additional file 4), highlighting that this phenomenon requires both the loss of mouse TREM2 and the presence of human tau.

### TREM2 deficiency alters the microglial response to human tau

**Fig. 3:**
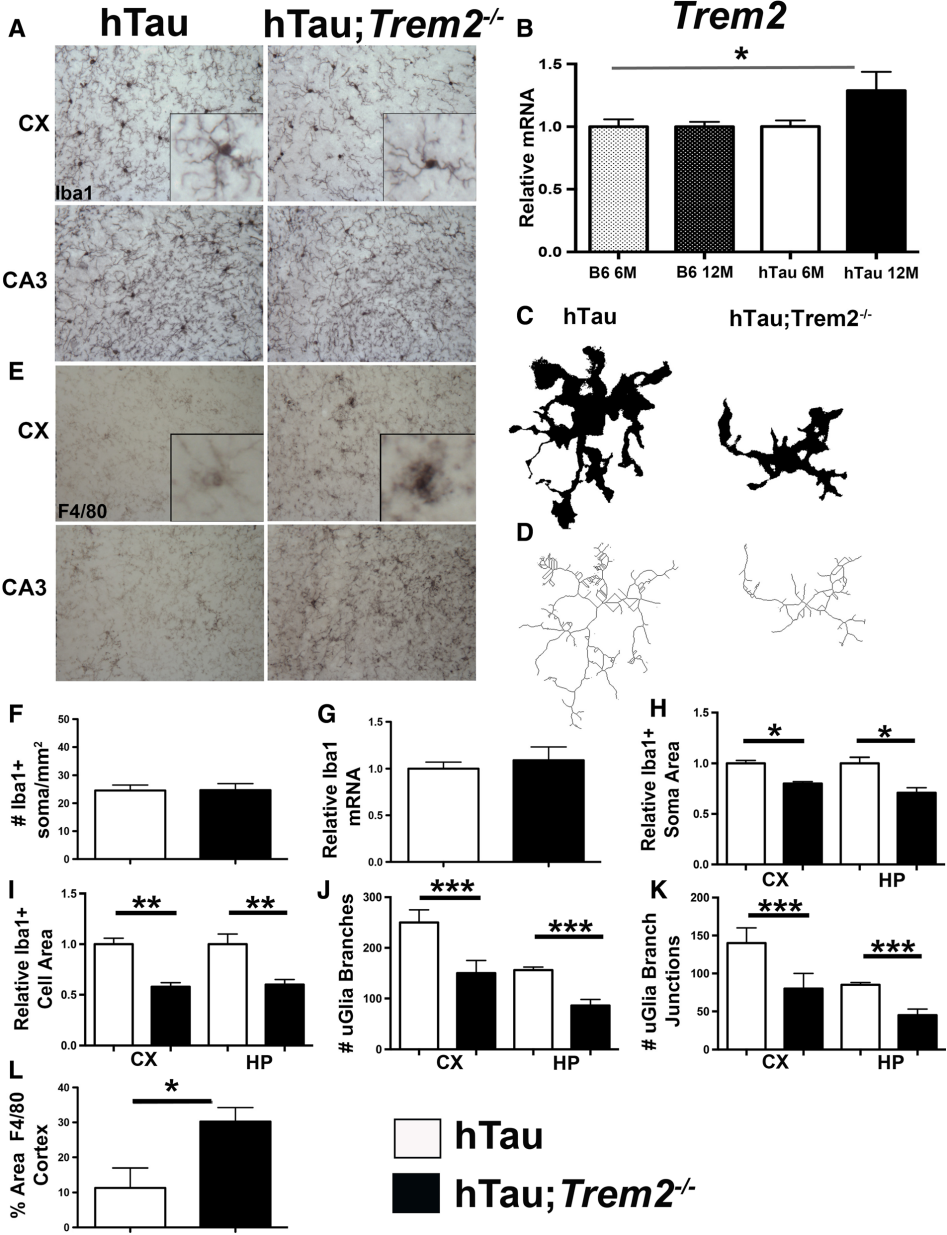
TREM2 deficiency leads to altered microglial activation. **a** IHC staining was performed on 30 μm thick sagittal sections from 6-month hTau (*Trem2* ^*+/+*^) and hTau;*Trem2* ^*−/−*^ mice (*n* = 6 per group; equal males and females) revealing altered morphological activation. **b** qRT-PCR was performed on hemi-brains from 6- and 12- month hTau (*Trem2* ^*+/+*^) and B6 control mice (*n* = 3–4 per group), revealing significantly upregulated TREM2 transcripts over time. **c** and **d** Individual microglia were selected randomly, isolated, thresholded, and skeletonized for analysis (20–30 per genotype) and compared between hTau and hTau;*Trem2* ^*−/−*^ mice within cortex and CA3 region of the hippocampus. (**e,l**) IHC staining was performed on hTau (*Trem2* ^*+/+*^) and hTau;*Trem2* ^*−/−*^ mice using antibodies against the pan-macrophage marker F4/80 (*n* = 6 per group). **f**, **g** Iba1 transcripts were analyzed from 6-month hTau (*Trem2* ^*+/+*^ *)* and hTau;*Trem2* ^*−/−*^ mice using qRT-PCR (*n* = 4–6 mice per group) alongside IHC staining with Iba1 (*n* = 6 per group) to perform a cell count which revealed no differences in the levels of Iba1 produced, or in the total number of cells/area **h-k** Detailed quantification of microglial morphology revealed decreased soma, cell area, branches, and branch points in TREM2 deficient mice compared to wild type hTau. At least two independent experiments were performed for each analysis. Error bars represent SEM., (**b,f,l**) Student’s *t*; (**h-k**) 2-way ANOVA with Sidak multiple comparisons correction) * *P* < 0.05. **, *P* < 0.01, ***, *P* < 0.001

To examine the expression of *Trem2* throughout the course of the development of tau pathology, we analyzed 12 month cohorts of non-transgenic B6 mice along with hTau transgenic mice. Quantitative RT-PCR analysis of B6 and hTau brains revealed a significant upregulation of *Trem2* transcripts in 12-month hTau mice compared to other experimental groups, consistent with a disease state dependent role for TREM2 in regulating tau pathology (Fig. 3b), analogous to that observed with amyloid pathology [28].

Previous studies demonstrated that microglia in hTau mice exhibit altered morphology that is accompanied by upregulation of expression of macrophage surface markers in an age and pathology dependent manner [29]. Immunohistochemistry of the microglial marker Iba1 was analyzed to determine the effect of TREM2 deficiency on microglial morphology in 6-month-old mice hTau mice (Fig. 3a). Microglia were randomly selected from the cortices and CA3 region of the hippocampi from hTau and hTau;*Trem2* ^*−/−*^ mice (Fig. 3c-l). hTau;*Trem2* ^*−/−*^ microglia were smaller overall (Fig. 3i) and cells exhibited thinner processes with significantly fewer branches and branch points (Fig. 3c–k) with relatively smaller soma areas (Fig. 3h), whereas microglia in age-matched hTau;*Trem2* ^*+/+*^ mice exhibited a more ramified appearance with extensive branching typical to the six-month time point. However, no differences were observed in either the total number of Iba1+ cells or in Iba1 transcript levels (Fig. 3f, g) between hTau;*Trem2* ^*+/+*^ mice and hTau;*Trem2* ^−/−^ mice at 6-months. Of note, microglia selected from the hippocampus trended towards significantly fewer branches and branch points than cortical microglia, which we can be attributed to the region of interest analyzed (CA3; Fig. 3). Importantly, non-transgenic and *Trem2* ^*−/−*^ microglia from 6-month control mice exhibited no significant morphological alterations (Additional file 4). To further characterize microglial phenotypes, brain sections were stained with antibodies against CD45 and F4/80, two macrophage surface markers that are chronically elevated throughout the course of many inflammatory neurodegenerative diseases, including tauopathies [30, 31]. Significant increases in F4/80 immunoreactivity were noted in the cortex and the CA3 region of the hippocampus in the brains of hTau;*Trem2* ^*−/−*^ mice when compared to hTau;*Trem2* ^*+/+*^ controls (Fig. 3e, l). By contrast, no differences were observed in CD45 expression throughout the brain in 6-month mice. Together, these studies demonstrate that TREM2 deficient hTau mice exhibit significant morphological and surface expression alterations in response to pathological human tau or deficiency in TREM2.

Increased expression of several genes characteristic of inflammation is a consistent feature of AD and related tauopathies, and includes alterations in genes historically termed “inflammatory” or “anti-inflammatory”. In order to explore a subset of these genes, the expression profile of the CNS in 6-month-old TREM2 deficient hTau mice, qRT-PCR was performed on whole brain lysates and a number of “pro- and anti-inflammatory” cytokines and chemokines, and other molecules associated with the microglial reaction were analyzed. No differences were observed in the transcript levels of IL-1β, iNOS, TNF-α, IL-6, TGF-β, RETNLB, ARG1, TLR4, or DAP12/TYROBP, a signaling adaptor for TREM2 (Additional file 5). Given the frequent and well reported disconnect between RNA and protein expression levels, we examined a number of canonical inflammatory markers and detected modest non-significant decreases in IL-1α, IL-1β, and IL-6 proteins (Additional file 5) with no differences detected in cortical or hippocampal lysates with regards to any other inflammatory mediators. Taken together, this line of investigation did not provide insight into the mechanism by which soluble and insoluble tau pathology was markedly enhanced in TREM2 deficient hTau mice.

### TREM2 deficiency leads to widespread dysregulation of stress signaling in hTau mice

**Fig. 4:**
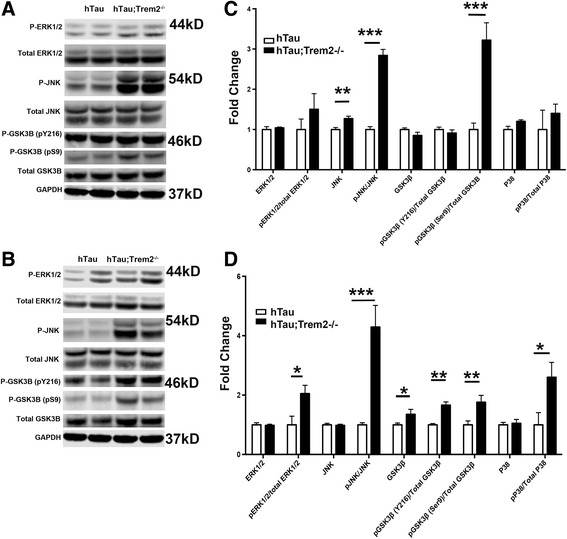
Increased activation of stress signaling molecules in TREM2 deficient hTau mice. **a-d** Western blot analysis was performed on microdissected cortices (**a**) and hippocampi (**b**) of hTau (*Trem2* ^*+/+*^) and hTau;*Trem2* ^*−/−*^ mice using antibodies directed against phosphorylated and total JNK, ERK1/2, GSK3β, and P38 to determine relative levels of MAPK activation. Quantification of western blot data revealed highly significant increases in total levels of JNK, as well as the ratio of pJNK/total JNK, and also very highly significant increases in the ratio pGSK3β(Ser9)/GSK3β in cortex (**c**), while robust increases in total levels of GSK3β were accompanied by significant increases in the ratios pERK1/2/total ERK1/2, pGSK3β(Ser9)/Total GSK3β, pGSK3β(Y216)/total GSK3β, pP38/total P38, and pJNK/total JNK in hippocampi of 6-month mice (**d**). All experiments used *n* = 6 (equal males and females) mice per group unless otherwise noted. At least two independent experiments were performed for each analysis. Error bars represent SEM. ANOVA with Tukey correction *, *P* < 0.05, **, *P* < 0.01, ***, *P* < 0.001

Signaling abnormalities in AD and related tauopathies include a wide variety of kinases that not only directly increase the pathological hyperphosphorylation of tau, but also oxidative stress and promote inflammation. Several kinases either directly phosphorylate tau or indirectly lead to increased phosphorylation of tau. Included in the group are the ERK, P38 and JNK families of MAP kinases as well as GSK3β. To address whether stress-related protein kinases might be implicated in the heightened pathology observed in hTau;*Trem2* ^*−/−*^ mice, Western blot analysis was performed. Strikingly, there were significant increases in total JNK within cortical samples (*P* < 0.01) as well as highly significant increases in the ratio of active pJNK/total JNK in both hippocampus and cortex (Fig. 4a-d; *P* < 0.001) in hTau;*Trem2* ^*−/−*^ mice when compared to hTau/*Trem2* ^*+/+*^ controls. Further, we detected robust increases in total GSK3β (Fig. 4b, d; *P* < 0.01) protein, along with significant increases in the ratios of active pGSK3β(Y216)/total GSK3β in the hippocampus (Fig. 4b, d; *P* < 0.01). Additionally, there were dramatically elevated levels of inactive pGSK3β(Ser9)/total GSK3β in hippocampus (Fig. 4b, d; *P* < 0.01) and cortex (Fig. 4a, c; *P* < 0.001) in TREM2 deficient hTau mice. Finally, significant increases in the ratios of active pERK1/2/total ERK1/2 and active pP38/total P38 were detected in the hippocampus of hTau;*Trem2* ^*−/−*^ mice (Fig. 4b, d; *P* < 0.05) when compared to controls, although the differences were modest in comparison to levels of JNK activation. Of important note, no abnormalities were detected among any of the previously analyzed signaling molecules in a control group of age-matched non-transgenic B6 or *Trem2* ^*−/−*^ mice (Additional file 4), again highlighting the requirement for TREM2 deficiency in combination with the presence of human tau.

Given the widespread signaling abnormalities observed in 6-month hTau;*Trem2* ^*−/−*^ mice, an additional cohort of 3-month-old animals was analyzed for early markers of signaling dysregulation. At 3 months of age there were no detectable differences in tau phosphorylation (Additional file 3). It was therefore unexpected that robust increases in total ERK1/2 and the ratio of pERK1/2/total ERK1/2 (P < 0.05), as well as marked and significant increases in the ratio of pJNK/total JNK were detected in hippocampi of TREM2 deficient hTau mice (P < 0.001; Additional file 3). Intriguingly, signaling alterations were absent in the cortex in 3-month mice. Taken together, these findings suggest that hippocampal kinase dysregulation in TREM2 deficient hTau mice occurs as early as 3 months of age. With time, this dysregulation is observed in other functionally connected brain regions and entrains additional kinases ultimately leading to exacerbated tau phosphorylation and aggregation at 6-months of age in hTau;*Trem2* ^*−/−*^ mice. These data also suggest that dysregulation of hippocampal JNK and ERK signaling represent early events that result in the exacerbated tau pathology seen in hTau;*Trem2* ^*−/−*^ mice, although exact mechanistic contributions of TREM2 deficient microglia in modifying tau pathology remain to be elucidated.

## Discussion

The current study demonstrates that TREM2 deficiency leads to worsened tau pathology, altered microglial reactivity, and robust signaling abnormalities in the hTau mouse model of tauopathy. These findings have direct implications for the role(s) of TREM2 in the clearance and/or propagation of tau pathology, in regulating Aβ and tau pathologies in opposing manners, in cell or activation specific (macrophage versus microglia) effects on AD pathologies and more broadly for neurodegeneration and AD as outlined below.

Our group and others previously characterized the effects of TREM2 deficiency in mouse models of AD that develop robust Aβ pathology. These studies consistently demonstrated that TREM2 deficiency leads to the loss of the majority of Aβ plaque associated macrophages throughout the diseased parenchyma. In addition to the altered myeloid cell accumulation, these studies demonstrated, an overall reduction in inflammatory markers, reduced reactive astrocytes and a reduction in tau phosphorylation within dystrophic neurites surrounding Aβ deposits. We also demonstrated TREM2 deficiency modestly reduces hippocampal Aβ pathology at 4 months of age. However, analysis of mice 8 months of age revealed an significant enhancement of amyloid burden in the TREM2 deficient mice in both the APPPS1 and 5XFAD mouse models of Aβ pathology [20, 24, 28]. However, neither the APPPS1 or 5XFAD mouse models of AD develop robust tau phosphorylation and aggregation (besides initial tau phosphorylation within the dystrophic neurites surrounding Aβ deposits). By contrast, the current study demonstrates that TREM2 deficiency results in robustly enhanced intraneuronal tau phosphorylation, aggregation, and kinase dysregulation in the hTau mouse model of tauopathy, suggesting opposing roles in modulating the two cardinal hallmarks of AD pathology.

We have demonstrated that TREM2 deficient microglia exhibit an altered morphology and surface expression profile in response to pathological human tau, which is consistent with other studies using various disease models. Here, we demonstrate a novel mechanism that exacerbates tau pathology independent of the up-regulation of classic pro-inflammatory mediators.

Our studies suggest that TREM2 plays a role in the regulation of JNK, GSK3β and ERK signaling and subsequent downstream tau hyperphosphorylation and aggregation. Taken together with our data demonstrating that reactive microglia localize to regions heavily laden with hyperphosphorylated tau suggests that TREM2 deficient microglia could fail to mount an efficient immune response in reaction to pathological tau accumulation, including potential deficiencies in clearance or phagocytosis of toxic tau, which is consistent with previous studies from the Landreth lab [12]. Hyperphosphorylation of GSK3β at both the activating Y216 and inactivating S9 epitopes may indicate an attempt by neurons to halt over activation of signaling, as GSK3β(S9) phosphorylation acts to negatively regulate GSK3β activity. This phenomenon could also be due to over-activity of additional upstream kinases that affect multiple signaling molecules at various phosphorylation sites. Additional studies are required to examine the nature of the signal that promotes kinase dysregulation in TREM2 deficient mice, including the timing, cell autonomous and cell non-autonomous mechanisms, and longer-term consequences with aging. Additionally, in-vivo pharmacological blocking agents against JNK, ERK, and GSK will be necessary to further elucidate the exact mechanism by which tau is being phosphorylated, but these studies are beyond the scope of the current report.

Several recent studies have demonstrated the spread of pathological tau into neighboring brain regions throughout the course of pathology via mechanisms which are currently poorly understood. Intriguingly, recent studies by Ikezu and colleagues demonstrated that ablation of microglia or inhibition of exosomal trafficking reduces tau pathology, suggesting that the observed spread of tau may potentially be mediated by microglia [32]. The observed hippocampal signaling abnormalities detected in 3-month hTau;*Trem2* ^*−/*−^ mice in combination with the localization of pathology in older 6-month mice leads us to posit that pathological tau and/ or signaling dysregulation spreads from the hippocampus into neighboring cortical regions, where the most prominent pathology is localized in hTau;*Trem2* ^*−/−*^ mice. The failure to effectively clear or process tau species from diseased neurons or the extracellular environment via normal clearance mechanisms could lead to further downstream kinase activation resulting in the eventual seeding and spread of pathological tau species from cell to cell and into different brain regions, giving explanation to our results which demonstrate soluble and insoluble tau pathology is worse in the cortex. These ideas are further supported by recent in-vitro studies which demonstrated that increased neuronal stress kinase signaling leads to increased presence of extracellular tau, although the exact mechanisms remain unclear [33]. However, future studies will be required to examine whether TREM2 deficiency promotes tau pathology via altered clearance and pathological spread of tau, direct microglial-neuronal contact and/or other possible mechanisms.

Taken together with our previous findings, the current study suggests that TREM2 deficiency has opposing roles on Aβ and tau pathologies, with an amelioration of Aβ-associated pathologies and yet an exacerbation of tau pathology. Several other studies of specific immune pathways have also demonstrated a similar opposing relationship on the development of Aβ and tau pathologies including the neuronal-specific chemokine, fractalkine (CX3CL1) and its cognate microglial receptor (CX3CR1) as well as the cytokine interleukin 1β (IL1β), amongst others [34, 35]. This accumulating evidence suggests that TREM2 and other innate immune pathways may play detrimental roles in early stages of human AD, when Aβ predominates (estimated to be as many as 10–20 years prior to clinical onset), but beneficial roles at later stages of human AD, when tau pathology develops, that has direct implications for the development of therapeutic strategies targeting these pathways. Furthermore, these results also suggest that innate immune pathways could potentially mechanistically link the two primary pathological hallmarks of AD, albeit in opposing manners. There are several different experimental models via which this might occur, including frustrated phagocytosis, altered clearance/propagation of extracellular/intracellular protein aggregates by myeloid cells and alternative roles of brain resident microglia and macrophages versus peripherally derived macrophages in regulating AD pathologies, which will need to be examined in future studies.

## Conclusions

In summary, our findings demonstrate a critical role for TREM2 in regulating tau related CNS innate immune responses, and identifies that TREM2 signaling plays a protective role in tauopathies despite evidence that signaling plays some detrimental roles in early Aβ pathological outcomes. It should be noted that investigators of TREM2 deficiency have employed several different varieties of TREM2 mutant knock-out mouse strains which may result in variabilities. These could be influenced by potential dysregulation of other genes within the locus and other compensatory mechanisms. Thus, parallel studies utilizing mice and cell models harboring the AD and FTD risk mutations including the R47H, Y38C, and T66 M TREM2 mutants, in addition to cell specific floxed TREM2 models, will be critical to directly assess the role of TREM2 in modifying tauopathy. Finally, given the results of recently published findings demonstrating potent signaling capacity of the soluble cleaved TREM2 protein, it will be necessary to explore the role of TREM2 cleavage in modifying tauopathy [36].

## Additional files

Additional file 1: Increased tau pathology in 6-month hTau;*Trem2−/−* mice. **A** Western blot shows increased conformation specific anti-tau antibody MC1 reactivity in hTau;*Trem2* ^*−/−*^ compared to hTau controls. **B** Increased ratio of Iba1 reactivity to AT8 Tau reactivity within cortices of hTau;*Trem2* ^*−/*−^ mice. At least two independent experiments were performed for each analysis, *n* = 4–6 mice per genotype; equal males and females. Error bars represent SEM. *, *P* < 0.05, **, *P* < 0.01, ***, *P* < 0.001. (PDF 956 kb)

Additional file 2: Quantification strategy for p-tau + neurons. **A** Cresyl violet staining was utilized to determine specific laminar layers II-III and IV-VI which were used to define quantitation of p-Tau^+^ neurons. A total of 2- medial sections were analyzed per mouse (*n* = 4 per genotype). Individual AT8 and AT180 positive cell bodies were counted in layers II-III and layers IV-VI in hTau and hTau;*Trem2* ^*−/−*^ mice. **B** No genotype specific differences in neurodegeneration were detected between hTau and hTau;*Trem2* ^*−/−*^ mice at 6 months of age as measured by NeuN reactivity. (PDF 1673 kb)

Additional file 3: MAPK signaling changes detected in 3- month hTau;*Trem2* ^*−/−*^ hippocampi despite no differences in tau pathology. Western blot analysis and quantification of hippocampal protein extracts from 3-month hTau (*Trem2* ^*+/+*^) and hTau;*Trem2* ^*−/−*^ mice (*n* = 4–6 per group) reveals significant upregulation of ERK1/2 and significant differences in the ratio of pERK1/2/total ERK1/2, and pJNK/total JNK. A reduction in GSK3β was observed in TREM2 deficient mice which lead to significant increases in the ratio of pGSK3β/total GSK3β, although no significant differences were observed between hTau and hTau;*Trem2* ^*−/−*^ mice with regard to the total levels of activated GSK3β. Cortices **(E,F)** from 3-month hTau (*Trem2* ^*+/+*^) and hTau;*Trem2* ^*−/−*^ mice (*n* = 4–6 per group) were analyzed using western blot. Quantification of these data revealed no significant differences between genotypes at 3-months in the cortex. At least two independent experiments were performed for each analysis, *n* = 4–6 mice per genotype; equal males and females. Error bars represent SEM. *, *P* < 0.05, **, *P* < 0.01, ***, *P* < 0.001. (PDF 9791 kb)

Additional file 4: **A,B** Western blot analysis reveals no alterations in cortical or hippocampal signaling molecules between 6-month non-transgenic BL6 control mice and *Trem2* ^*−/−*^ mice. Additionally, no significant alterations in p-tau **(C)** were detected between BL6 and *Trem2* ^*−/−*^ genotypes. Modest non-significant increases in total tau (Tau5) were detected in *Trem2* ^*−/−*^ mice compared to BL6 non-transgenic mice. **(D)** Morphological analysis of Iba1 staining demonstrates no alterations among microglia between BL6 and *Trem2* ^*−/−*^ control mice (*N* = 4 mice per genotype). At least two independent experiments were performed for each analysis. Error bars represent SEM. *, *P* < 0.05, **, *P* < 0.01, ***, *P* < 0.001. (PDF 1980 kb)

Additional file 5: Quantitative RT-PCR and Cytokine multiplex analysis of 6-month hTau and hTau;*Trem2* ^*−/−*^ mice. **A** Whole-brain lysates from 6-month hTau (*Trem2* ^*+/+*^) and hTau;*Trem2* ^*−/−*^ mice (*n* = 5–6 per group) were prepared for analysis using qRT-PCR with primers directed against numerous pro- and anti-inflammatory transcripts using the ΔΔCT method, and normalized to hTau levels. **B** Cytokine/chemokine multiplex assays were performed on dissected cortex and hippocampus in 6-month hTau (*Trem2* ^*+/+*^) and hTau;*Trem2* ^*−/−*^ mice (*n* = 10 per group). At least 2 independent experiments were performed for each analysis. (PDF 3397 kb)
