# Supplementary figures and images for: TREM2 deficiency exacerbates tau pathology through dysregulated kinase signaling in a mouse model of tauopathy
Source: Mol Neurodegener. 2017 Oct 16;12:74. doi: 10.1186/s13024-017-0216-6 (PMC5644120; doi:10.1186/s13024-017-0216-6)

**A****CX**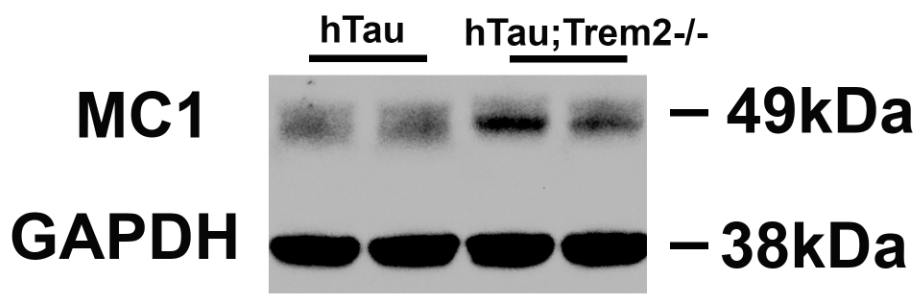**MC1/Tau5**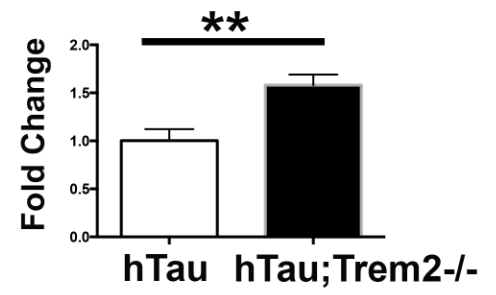**B**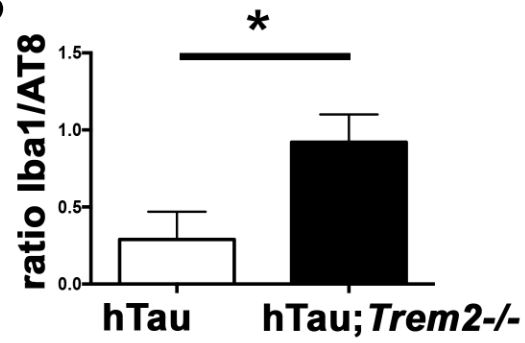

Supplement: Supplementary file 1 — Increased tau pathology in 6-month hTau;Trem2−/− mice. A Western blot shows increased conformation specific anti-tau antibody MC1 reactivity in hTau;Trem2 −/− compared to hTau controls. B Increased ratio of Iba1 reactivity to AT8 Tau reactivity within cortices of hTau;Trem2 −/− mice. At least two independent experiments were performed for each analysis, n = 4–6 mice per genotype; equal males and females. Error bars represent SEM. *, P < 0.05, **, P < 0.01, ***, P < 0.001. (PDF 956 kb) [file 13024_2017_216_MOESM1_ESM.pdf]

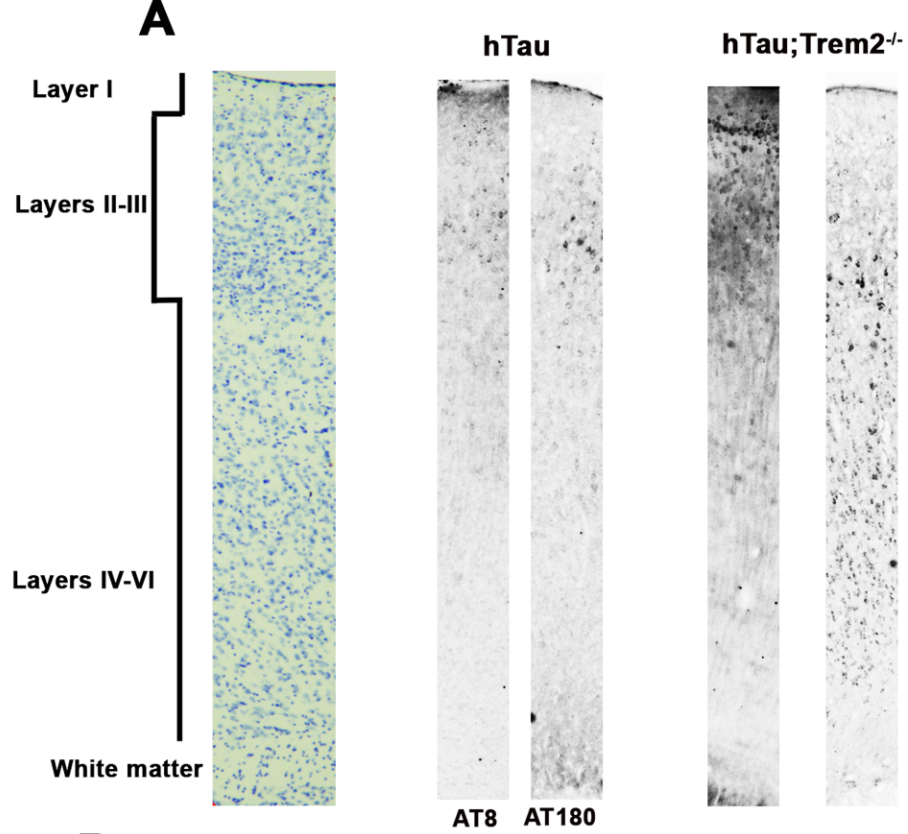

**B**

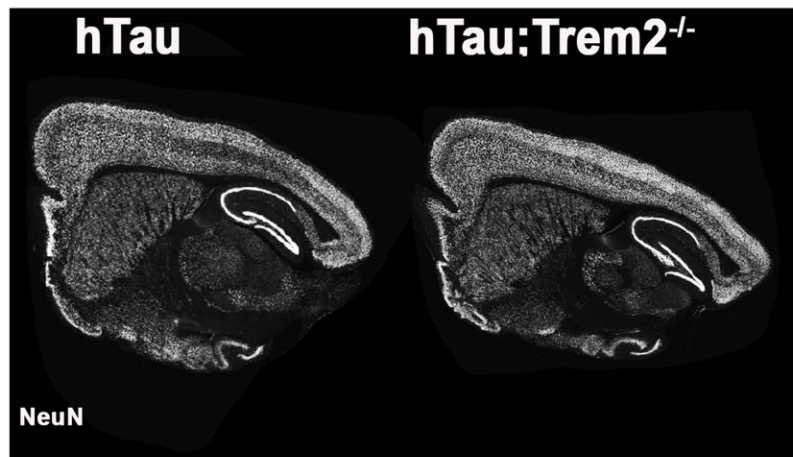

Supplement: Supplementary file 2 — Quantification strategy for p-tau + neurons. A Cresyl violet staining was utilized to determine specific laminar layers II-III and IV-VI which were used to define quantitation of p-Tau+ neurons. A total of 2- medial sections were analyzed per mouse (n = 4 per genotype). Individual AT8 and AT180 positive cell bodies were counted in layers II-III and layers IV-VI in hTau and hTau;Trem2 −/− mice. B No genotype specific differences in neurodegeneration were detected between hTau and hTau;Trem2 −/− mice at 6 months of age as measured by NeuN reactivity. (PDF 1673 kb) [file 13024_2017_216_MOESM2_ESM.pdf]

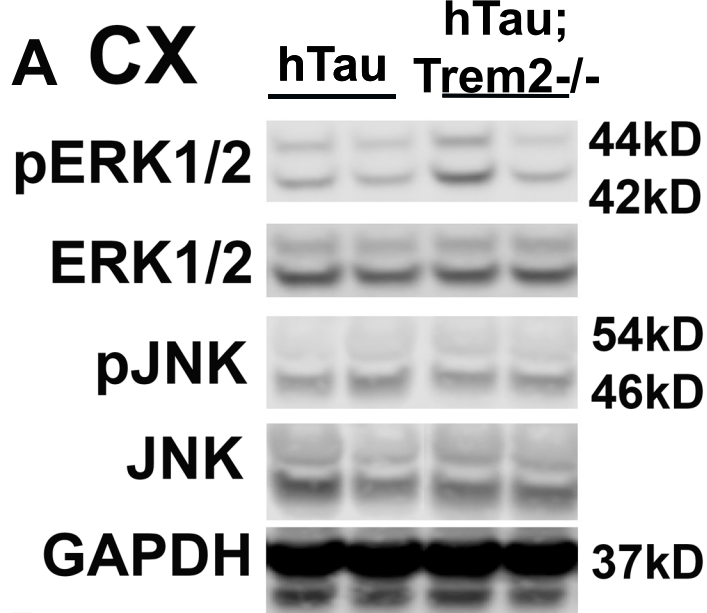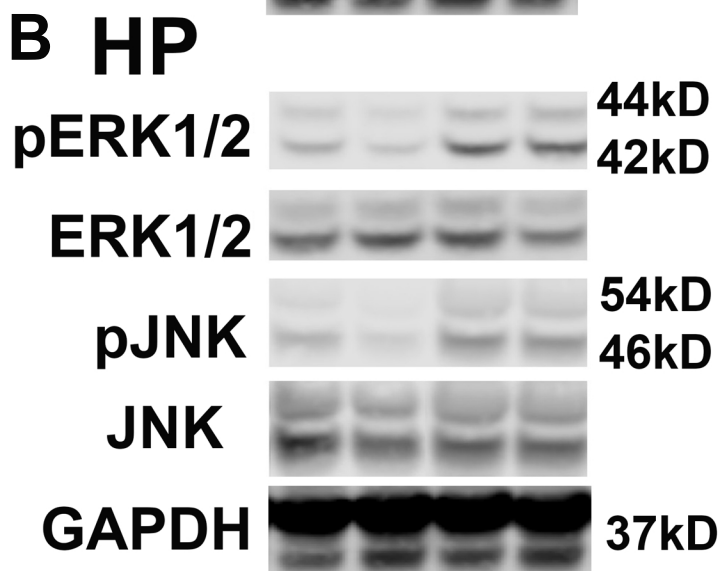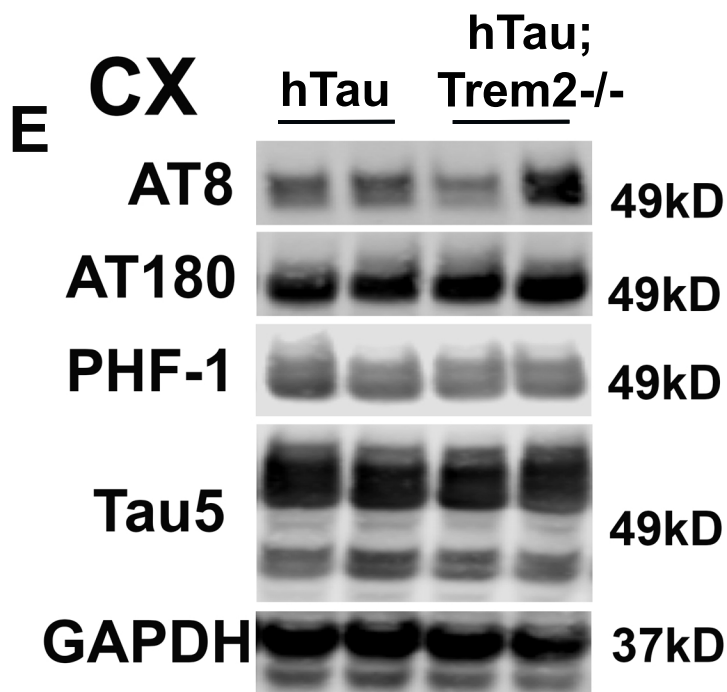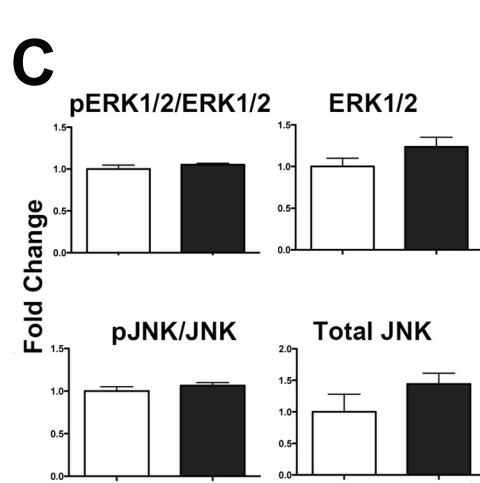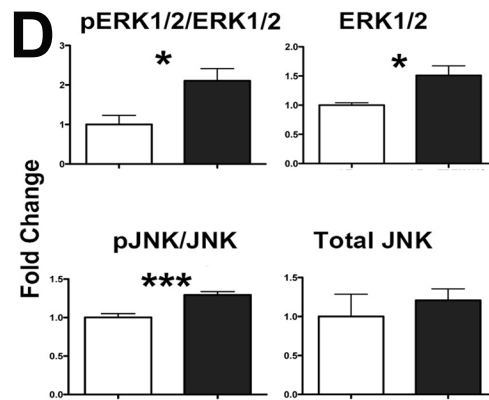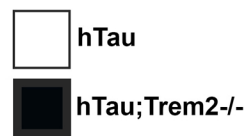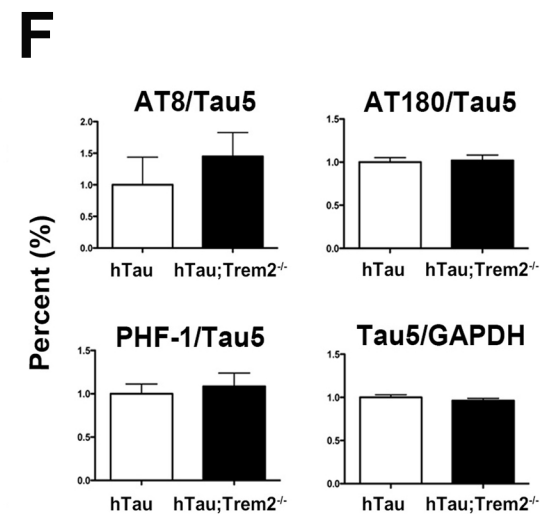

Supplement: Supplementary file 3 — MAPK signaling changes detected in 3- month hTau;Trem2 −/− hippocampi despite no differences in tau pathology. Western blot analysis and quantification of hippocampal protein extracts from 3-month hTau (Trem2 +/+) and hTau;Trem2 −/− mice (n = 4–6 per group) reveals significant upregulation of ERK1/2 and significant differences in the ratio of pERK1/2/total ERK1/2, and pJNK/total JNK. A reduction in GSK3β was observed in TREM2 deficient mice which lead to significant increases in the ratio of pGSK3β/total GSK3β, although no significant differences were observed between hTau and hTau;Trem2 −/− mice with regard to the total levels of activated GSK3β. Cortices (E,F) from 3-month hTau (Trem2 +/+) and hTau;Trem2 −/− mice (n = 4–6 per group) were analyzed using western blot. Quantification of these data revealed no significant differences between genotypes at 3-months in the cortex. At least two independent experiments were performed for each analysis, n = 4–6 mice per genotype; equal males and females. Error bars represent SEM. *, P < 0.05, **, P < 0.01, ***, P < 0.001. (PDF 9791 kb) [file 13024_2017_216_MOESM3_ESM.pdf]

# 6-month

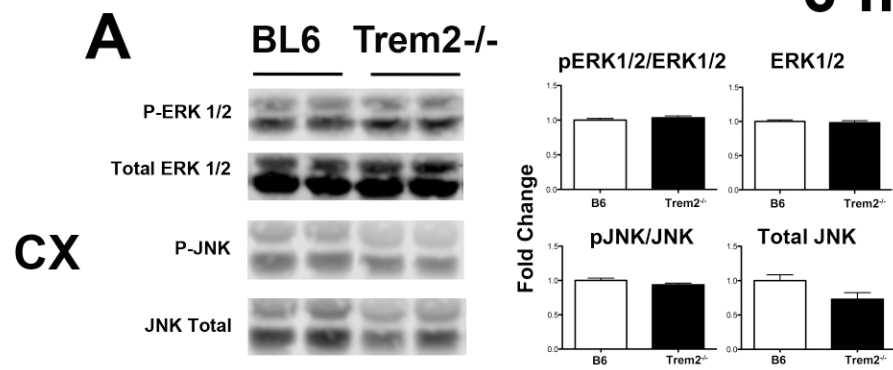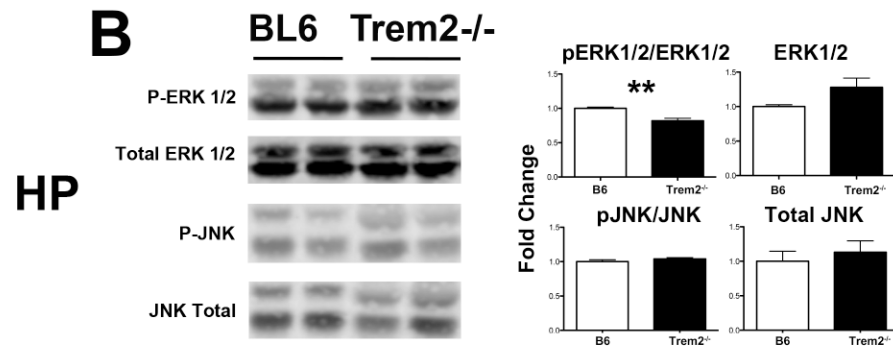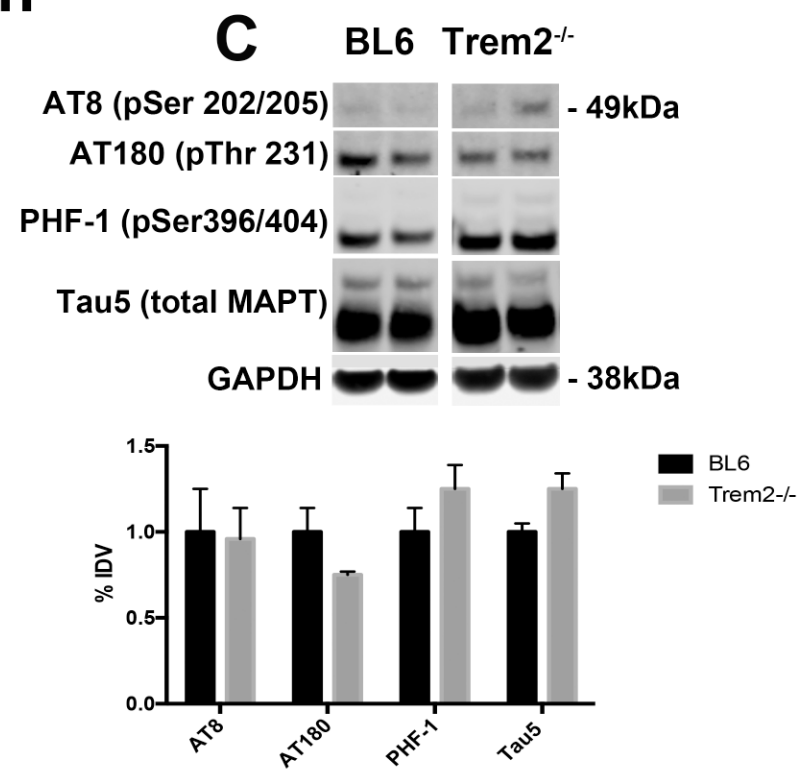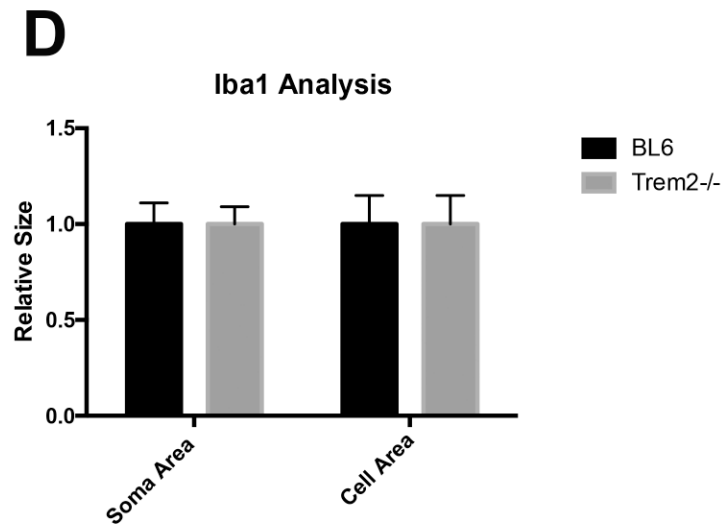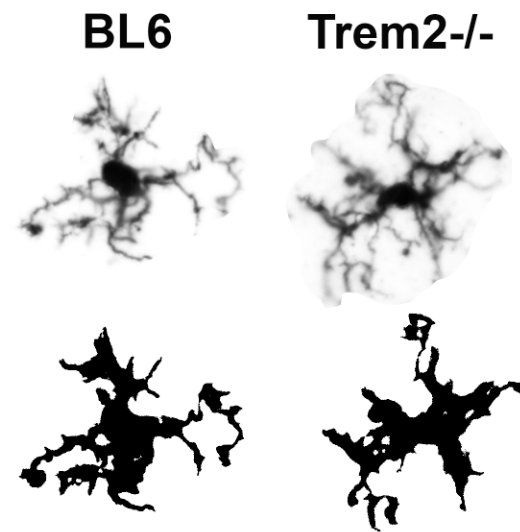

Supplement: Supplementary file 4 — A,B Western blot analysis reveals no alterations in cortical or hippocampal signaling molecules between 6-month non-transgenic BL6 control mice and Trem2 −/− mice. Additionally, no significant alterations in p-tau (C) were detected between BL6 and Trem2 −/− genotypes. Modest non-significant increases in total tau (Tau5) were detected in Trem2 −/− mice compared to BL6 non-transgenic mice. (D) Morphological analysis of Iba1 staining demonstrates no alterations among microglia between BL6 and Trem2 −/− control mice (N = 4 mice per genotype). At least two independent experiments were performed for each analysis. Error bars represent SEM. *, P < 0.05, **, P < 0.01, ***, P < 0.001. (PDF 1980 kb) [file 13024_2017_216_MOESM4_ESM.pdf]

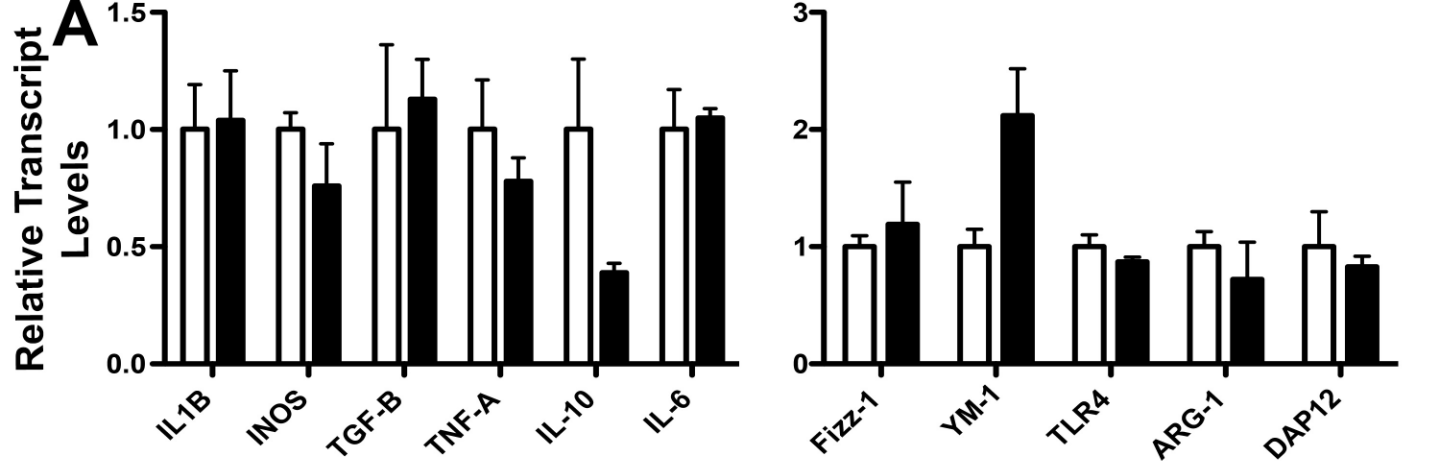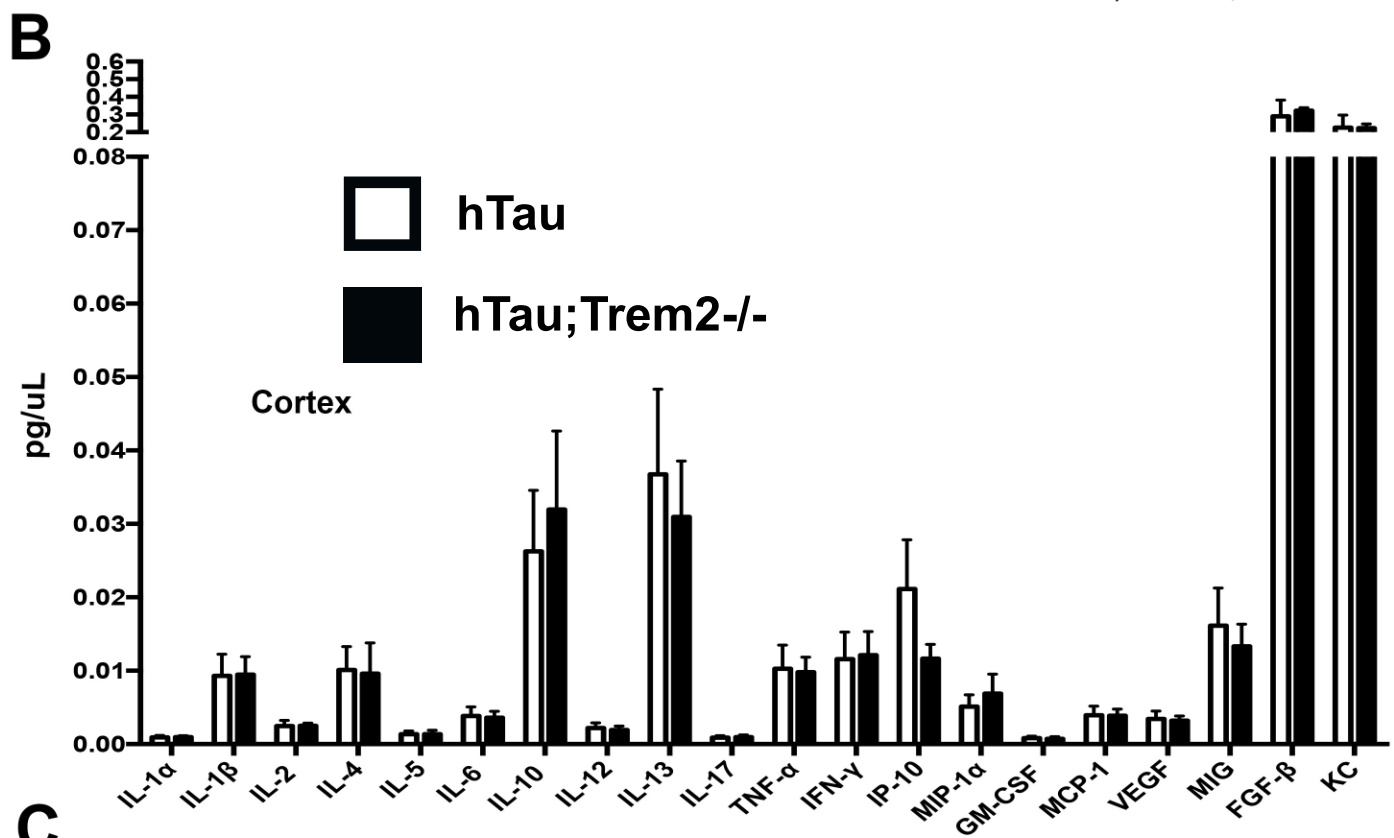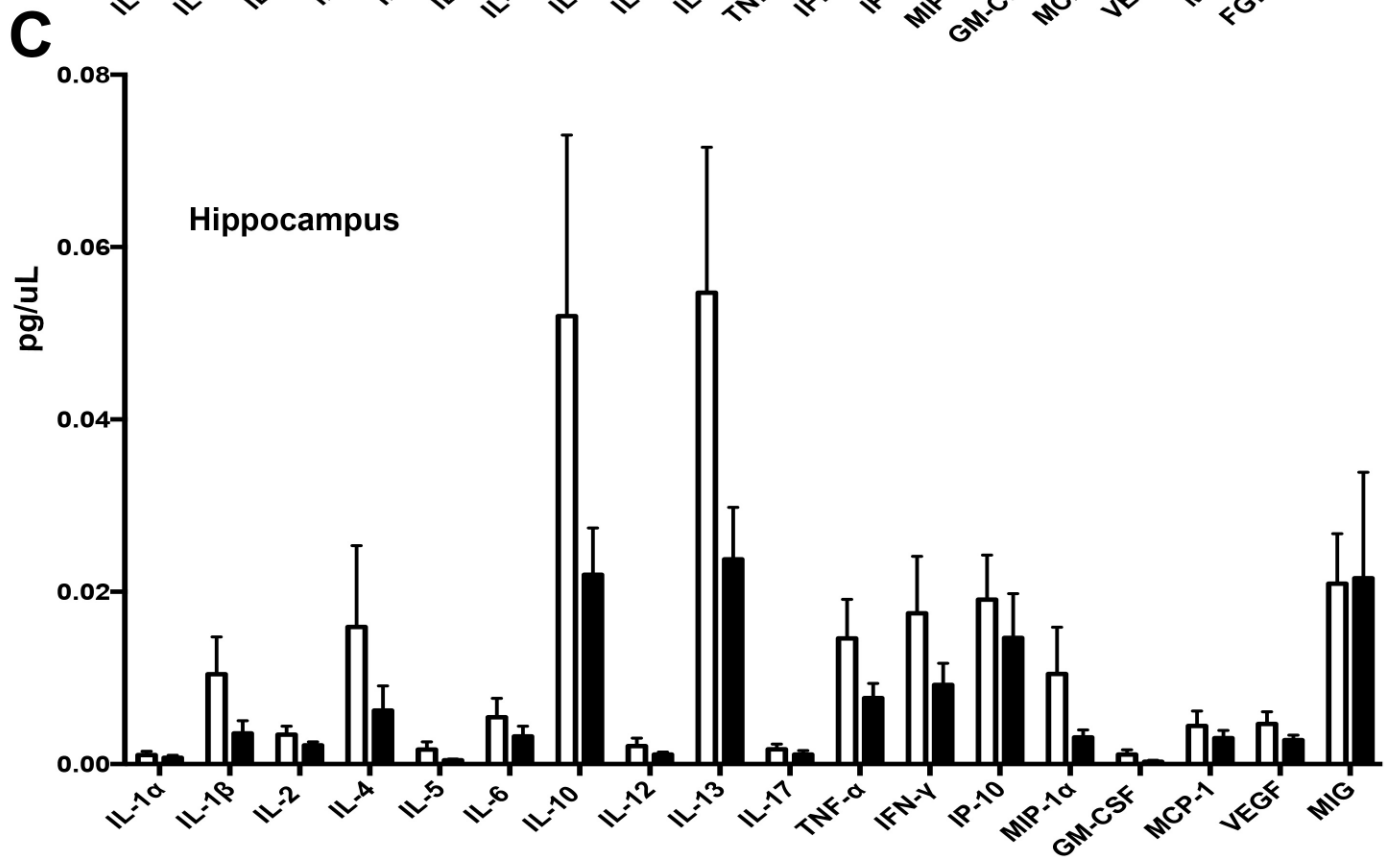

Supplement: Supplementary file 5 — Quantitative RT-PCR and Cytokine multiplex analysis of 6-month hTau and hTau;Trem2 −/− mice. A Whole-brain lysates from 6-month hTau (Trem2 +/+) and hTau;Trem2 −/− mice (n = 5–6 per group) were prepared for analysis using qRT-PCR with primers directed against numerous pro- and anti-inflammatory transcripts using the ΔΔCT method, and normalized to hTau levels. B Cytokine/chemokine multiplex assays were performed on dissected cortex and hippocampus in 6-month hTau (Trem2 +/+) and hTau;Trem2 −/− mice (n = 10 per group). At least 2 independent experiments were performed for each analysis. (PDF 3397 kb) [file 13024_2017_216_MOESM5_ESM.pdf]
